# Unified Camera Tamper Detection Based on Edge and Object Information

**DOI:** 10.3390/s150510315

**Published:** 2015-05-04

**Authors:** Gil-beom Lee, Myeong-jin Lee, Jongtae Lim

**Affiliations:** 1 School of Electronics, Telecommunication & Computer Engineering, Korea Aerospace University, 76 Hanggongdaehak-ro Deogyang-gu, Goyang-si, Gyeonggi-do 412-791, Korea; E-Mail: lgbch2@gmail.com; 2 School of Electronic & Electrical Engineering, Hongik University, 94 Wausan-ro, Mapo-gu, Seoul 121-791, Korea; E-Mail: jlim@hongik.ac.kr

**Keywords:** camera tamper detection, edge disappearance rate, adaptive threshold, covered event, moved event, defocused event

## Abstract

In this paper, a novel camera tamper detection algorithm is proposed to detect three types of tamper attacks: covered, moved and defocused. The edge disappearance rate is defined in order to measure the amount of edge pixels that disappear in the current frame from the background frame while excluding edges in the foreground. Tamper attacks are detected if the difference between the edge disappearance rate and its temporal average is larger than an adaptive threshold reflecting the environmental conditions of the cameras. The performance of the proposed algorithm is evaluated for short video sequences with three types of tamper attacks and for 24-h video sequences without tamper attacks; the algorithm is shown to achieve acceptable levels of detection and false alarm rates for all types of tamper attacks in real environments.

## 1. Introduction

Recently, video analytics has been widely deployed in various application areas, including video surveillance, business intelligence and the Internet of Things [1–7]. Especially in video surveillance systems, intelligent video analytics can reduce the cost of video monitoring and increase the surveillance system performance by automatically analyzing video content to detect a variety of events, such as intrusions, violence, fire, camera tamper attacks, and so on. A camera tamper attack is an event that disturbs the normal camera capturing process for malicious purposes; thus, camera tamper detection is an essential task in implementing video surveillance systems.

The variety of camera tamper attacks can be classified into three types: defocused, covered and moved camera events [8–10]. A defocused camera event is one in which the focal length of the camera is changed to cause blurring of the captured video; a covered camera event is one in which the camera lens is partially or totally occluded by external objects; a moved camera event refers to a situation in which the camera viewing angle is abruptly changed by external forces or the camera is titled by wind or an earthquake. Camera tamper attacks usually alter the characteristics of the captured images, which we can exploit to detect such an attack. For example, a defocused camera event causes power reduction of the high frequency components in the transform domain or the edges of the captured image; a covered camera event changes the distribution of the image intensity histogram or the image edges.

Most previous studies on tamper detection have compared features of the current frame with those of the background frames [8,10–12]. Features extracted from an image frame include the intensity histogram, edge map and high frequency components after the wavelet transform (WT), the discrete Fourier transform (DFT) or the discrete cosine transform (DCT). Because the background frame can reflect changing environmental conditions gradually, it can be a stable reference for comparison. Other studies have used features only from the current frames, without using the background frame for comparison; these methods have used histograms of chromaticity, intensity and saturation [13], a blur measure based on the Sobel edge [14] and global and local edge energy [9]. Although these studies have allowed the saving of some resources for background frame generation, these algorithms have high false alarm rates, because the utilized features of the current frame or the threshold parameters cannot reflect the time-varying characteristics of the scene.

These studies have used different features and algorithms to detect each tamper event. Although a unified tamper detection criterion was proposed in our previous study [15], based on the edge changed rate, and in [9], based on global and local edge energies, the methods used in these studies were still susceptible to scene dynamics. Furthermore, most studies have used quite many thresholds of features for tamper detection, which makes it difficult for the algorithms to be applied to different environmental conditions with optimally-configured thresholds [8,9,11–13,16]. In terms of performance, although most studies have tried to reduce the false alarm rate, experiments were performed only for very short video sequences, which cannot reflect the real video surveillance environment, which has such characteristics as illumination change over 24-h period, scene dynamics, *etc*. Furthermore, there is no measure that can be used for fair comparison of the false alarm rate among tamper detection algorithms. Therefore, it is required to design a unified tamper detection algorithm with a small number of features that is robust against scene dynamics. It is also required to provide a way to measure the false alarm rate and to allow performance evaluation for sufficiently long video sequences.

In this paper, a novel unified tamper detection algorithm is proposed to detect all types of camera tamper attacks from video sequences with scene dynamics based on object and edge information in video sensor networks or IP camera-based video surveillance environments. Because most video sensor nodes and IP cameras act as input sources for video analytics modules residing on themselves or for remote servers that may be the video data sinks, taking advantage of video analytics information, such as information of objects and the background, can greatly reduce the computational complexity of tamper detection. In this study, to tackle the false alarm problem of scene dynamics, edge information and object information extracted from video analytics modules are used to detect camera tamper attacks without using the computationally-intensive transforms and filters used in conventional studies. Object removal for edge extraction and adaptive thresholds for different scene and illumination characteristics are proposed to reduce the false alarm rate and to enhance the accuracy of tamper detection, respectively. Furthermore, in order to maintain the tamper attack alerts until an operator's investigation and cancellation, background absorption of tamper events after the occurrence of tamper events is prevented.

The organization of this paper is as follows. In Section 2, the architecture of the video analytics algorithms is proposed; this architecture is suitable for the proposed camera tamper detection algorithm. In Section 3, we propose a unified camera tamper detection algorithm based on background frame and object information from the video analytics algorithms. In Section 4, we present experimental results for the proposed algorithm with real daytime and nighttime video sequences, including camera tamper events. In Section 5, we present our conclusion and suggestions for further work.

## 2. Architecture of Video Analytics Systems with Camera Tamper Detection

Most conventional studies have used a background frame as a reference for comparison with a current frame [8,10–12]. Although background generation is computationally intensive, because every pixel in a frame should keep track of its own statistical or temporal background models, tamper detection algorithms using background and current frames have shown performance better than that of other algorithms using features only from the current frame [8–14]. This is because the background frame can reflect the changing environmental conditions gradually.

**Figure 1:**
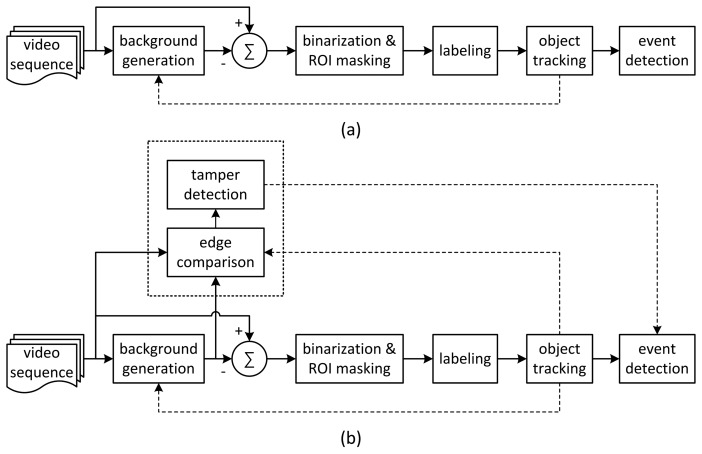
Camera tamper detection in video analytics systems: (**a**) video content analytics algorithm; (**b**) video content analytics algorithm with proposed tamper detection.

A conventional video analytics algorithm based on background subtraction is shown in Figure 1a [3,6]. To extract foreground objects, background generation, binarization and labeling are performed on input images. Finally, object tracking and predefined event detection are performed.

In this study, we attempt to detect all types of camera tamper events with a unified criterion that can be embedded into video analytics algorithms based on background subtraction. As a common and unified feature to detect covered, defocused and moved events, edge change information between current and background frames is used. To decrease the false alarm rate that results from scene dynamics, object information from the video analytics algorithm is used to prevent foreground edges from being used for detection. Figure 1b shows the proposed architecture of a novel video analytics algorithm with tamper detection in which the object information and background frames can be taken from the results of the video analytics algorithm. The Gaussian mixture model (GMM) is used for the background generation, because it can gradually update background images without abrupt changes [17]. Background subtraction is used for foreground extraction, and contour labeling is used to obtain object information from the foreground pixels. Small labeled objects of less than a certain threshold in size are considered to be noise blobs and are removed. The results of camera tamper detection can help in main event detection for such events as intrusions, traffic collisions and loitering in the video analytics algorithm to suppress wrong events caused by camera tamper attacks. Although this architecture requires additional computational complexity for edge calculation, the increase of the complexity is negligible compared with that of the video analytics algorithm.

## 3. Unified Camera Tamper Detection Based on Edge and Object Information

### 3.1. Signal Characteristics of Camera Tamper Attacks

For camera tamper detection, it is necessary to find common features and signal characteristics in covered, defocused and moved events. For covered and defocused events, captured image frames get simpler and finally lose their high frequency components. For moved events, due to the changing or changed camera angles, captured image frames may lose a large number of high-frequency components due to the motion blurring effect during a camera moving period or the number of high frequency components at the final moved angle may be quite different from that in the initial angle.

Conventional studies of tamper detection have focused on the decreasing tendency of high-frequency components in input image frames after tamper attacks [8,9,11–13,16,18]. These studies have tried to separate detection criteria for each type of tamper attack using features, such as edge energy, or high-frequency components from the DFT, the WT or the scale-invariant feature transform (SIFT), which have large computational loads. However, because quite a few thresholds of features were used, it was difficult to find optimal thresholds for different environmental conditions.

**Figure 2:**
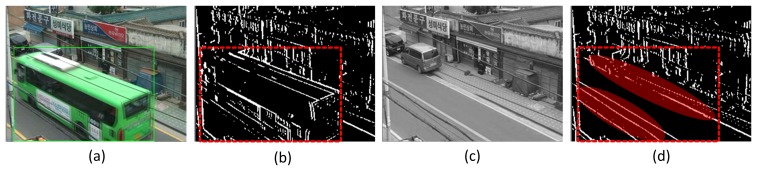
Difference of edge pixels between current and background frames due to scene dynamics: (**a**) current image; (**b**) current edge image; (**c**) background image; (**d**) background edge image.

In tamper detection, scene dynamics, such as the moving of foreground objects or time-varying illumination conditions, may affect the accuracy and false alarm rate of tamper detection. Conventional studies have suffered from performance degradation due to scene dynamics [8–11,14,16]. If the background frame is generated, moving objects do not appear or only their dim vestige exists in background frames, as is shown in Figure 2c. In such cases, as can be seen in Figure 2b,d, if, after DFT, WT or SIFT, features, such as edges or high-frequency components in the background and current frames, are simply compared, false alarms may occur because the features of foreground objects are counted for tamper detection. False alarms may also occur for cases in which, inside the images, there are continuous movements of swaying trees, or ripples on the sea or on a river, or when there are noises. For time-varying illumination conditions, the features also may vary over time; features in daytime video sequences will be quite different from those in nighttime video sequences. Fixed thresholds for features may not be applicable to real surveillance or video sensor application environments with 24-h operation. Therefore, an adaptive decision threshold for features should be used to reflect this case.

### 3.2. Overview of the Proposed Tamper Detection Algorithm

**Figure 3:**
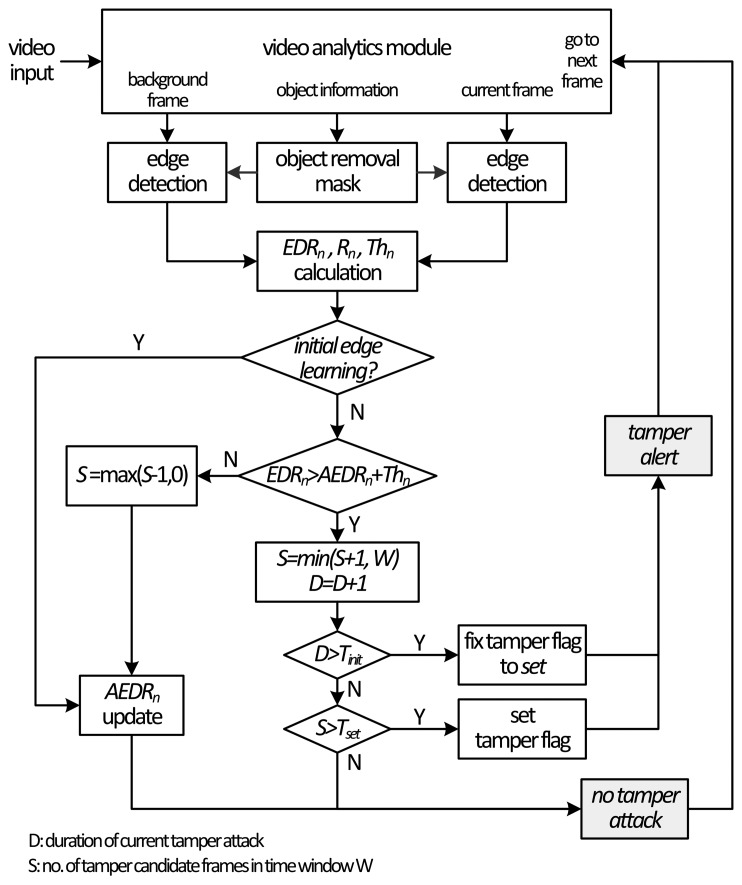
Proposed camera tamper detection algorithm with an adaptive threshold for a unified detection criterion.

The proposed tamper detection algorithm is shown in Figure 3. Tamper events are detected by the rate of the disappearance of edge pixels in the current frame compared to the edge pixels in the background frame. This rate is termed the edge disappearance rate (EDR). Several foreground objects in the current frame, if their sizes are not so big and the number of objects is not large, may not cause a big difference in the number of edge pixels in the current frame. However, big foreground objects, such as buses, or multiple foreground objects, sometimes occlude quite a large portion of the background in the current frame, as can be seen in Figure 2. If the edge characteristics of the foreground objects are quite different from those of the occluded regions, there will be an abrupt change in the number of pixels in the current frame; this situation can result in false alarms.

Therefore, the authors propose to use object information to exclude regions of objects in the background and current frames from the calculation of the EDR for tamper detection. The background frame is generated using GMM; foreground objects can be extracted by conventional background subtraction-based video analytics algorithms, as can be seen in Figure 1b.

In our previous study, a Canny edge detector was used to detect the edges for the current and background frames [15]. However, because of its edge thinning process, the Canny edge detector is computationally complex and not robust against the swaying of cameras or trees due to wind [19]. In the proposed algorithm, the Sobel filter is used for edge detection, because its computational load is quite a bit lower than that of the Canny edge detector, and the filtered result is less sensitive to the swaying of cameras or trees due to wind [20]. The object regions in the current and background frames are excluded from edge detection, as explained above.

### 3.3. Edge Disappearance Rate Excluding Foreground Regions

During a camera tamper attack, the number of edge pixels, *i.e.*, the high-frequency component of the current frame, tends to decrease. However, using only the edge information for detection may cause a false alarm for scene dynamics due to foreground objects. Therefore, the authors propose to use a measure that can exclude the regions of foreground objects; in this method, the object information can be taken from the video analytics algorithm.

To measure the amount of edge change when excluding foreground objects, the EDR for the current frame *n*, the ratio of the number of edge pixels that have disappeared in the current frame to the number of edge pixels in the background frame, is defined as follows.

(1) $${\text{EDR}}_{n}=1-\frac{\sum_{p\in R^{C}}e_{n,p}^{BG}\cdot e_{n,p}^{C}}{\sum_{p\in R^{C}}e_{n,p}^{BG}}$$

where $e_{n,p}^{C}$ and $e_{n,p}^{BG}$ represent with binary code the existence of an edge at pixel *p* in the current frame *n* and the background frame updated right before the frame *n*, respectively; values are one for an edge pixel and zero for a non-edge pixel. *R^C^* represents the region in the current frame without any foreground object; this region can be obtained from the object information provided by the video analytics algorithms.

**Figure 4:**
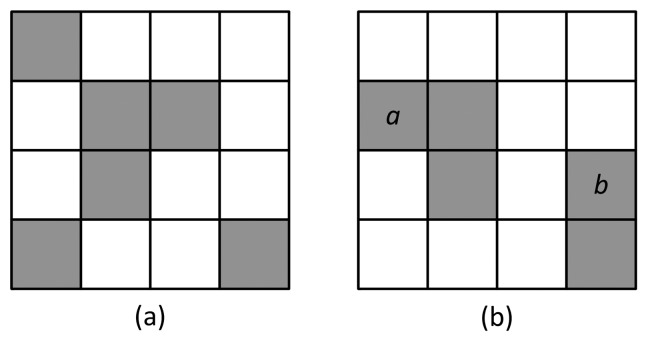
An example image for edge disappearance rate (EDR) calculation: (**a**) background edge image; (**b**) current edge image.

EDR represents the decreased number of edge pixels in the current frame, compared to the number of pixels in the background frame, due to camera tamper attacks. Figure 4 shows a simple example of EDR calculation. The white and gray pixels represent non-edge and edge pixels in the current and background edge pixel maps, respectively; maps are not overlapped with the foreground regions. The pixels *a* and *b* represent the edge of an object or noise existing only in the current image. For the 4 × 4-sized background and current images, $\sum_{p\in R^{C}}e_{n,p}^{BG}=6$, $\sum_{p\in R^{C}}e_{n,p}^{BG}\cdot e_{n,p}^{C}=3$ and *EDR_n_* = 0.5.

If the foreground region is quite complex, with many edges, only removing the edges in the current frame may greatly affect the EDR for non-tamper attacks. Therefore, for a fair comparison, an object removal mask should be applied to both the current and the background frames.

If camera tamper attacks occur, an entire image frame is sometimes detected as a single foreground object, because object detection is based on background subtraction. Such a situation may cause wrong EDR calculation by excluding entire edges of the image frame. Therefore, in our study, only objects less than a certain threshold in size are subjected to object removal. The threshold should be determined by considering the statistics of object information in the video sequences.

### 3.4. Background Absorption of Tamper Attacks

**Figure 5:**
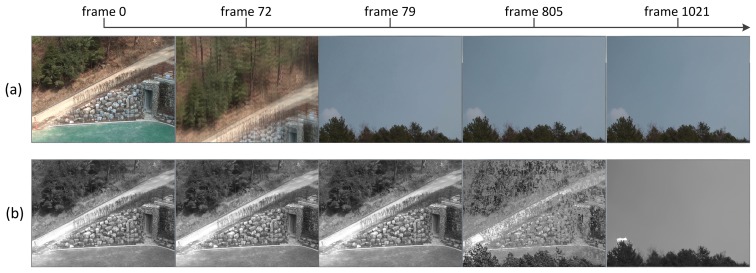
Background absorption of tamper attacks: (**a**) current image; (**b**) background image.

Although the background is updated by GMM gradually, if some changes in scene content are maintained in specific regions in the image frame or in the entire frame for a long period, the changes will be gradually absorbed into the background. Figure 5 shows an example of tampered image absorption into the background; a moved event occurs from Frame 72 and lasts until Frame 1021. The background frame gets changed from the one before the tamper attack to the tampered image frame. How fast the change of scene is absorbed into the background can be controlled by the learning rate γ in GMM. Table 1 summarizes the parameters for GMM used in our study.

General camera tamper attacks last long; *i.e.*, something sprayed on the camera keeps the camera from capturing a normal video sequence until the replacement or cleaning of the camera lens. Therefore, if the tamper attack is not handled quickly enough by the operator, the tampered region on the image frame may be absorbed into the background, and the tamper attack will not be detected.

To solve this problem, if the detected tamper attack lasts longer than a certain period, a tamper flag is fixed in such a way that it cannot be unfixed by the tampered background, as shown in Figure 3. Once the flag is fixed, the tamper alert lasts until it is cleared by the operator. The period for fixing the tamper flag is determined based on the learning rate of GMM.

### 3.5. Adaptive Threshold Reflecting Scene Dynamics

After object removal and EDR calculation for incoming image frames, a tamper decision should be made. Using an adaptive threshold, the proposed algorithm decides on the presence of a tamper attack by comparing the difference between the EDR and the average EDR.

During the initial period of tamper detection, the average EDR (AEDR) is calculated using the EDRs of the first *T_init_* frames. After this period, the AEDR is updated if no tamper attack is found to have occurred, as follows.

(2) $${\text{AEDR}}_{n}=\frac{1}{n-l}\sum_{m=1}^{n}(1-f_{m}^{t})\cdot {\text{EDR}}_{m}$$

where *n* and *l* represent the number of frames processed and the number of frames with tamper attacks until the current frame, respectively. $f_{m}^{t}$ is a tamper flag for the *m^th^* frame, with values of zero for no tamper and one for tamper events. If any tamper attack occurs, the AEDR keeps the current value.

The threshold should be adaptive to the time- or location-dependent illumination conditions or plain background. Because the level of EDR depends on the characteristics of the scene contents, the threshold for tamper detection should be adaptive to scene characteristics. Scene characteristics can be classified into four cases: normal, low illumination, large foreground region and plain background.

For the low illumination case, especially for nighttime sequences, although artificial lights or infrared (IR) capture capability are used to enhance the quality of the video sequences, extracted features for tamper detection have low reliability due to noise and low signal intensity. However, the proposed algorithm can exclude most noise from the EDR calculation without any anti-noise processing, because background frames generated by GMM include persistent signal changes that gradually exclude highly-variant noises. Because the number of edges in low illuminated frames tends to decrease due to low signal intensity, the level of EDR is generally lower than that in daytime video sequences. Edge changes due to instant changes of illumination are occasionally detected as objects and are excluded for EDR calculation by object removal.

For the case of large foreground region, there exist many foreground objects that can cover quite a large portion of the image frame; the region left for edge comparison provides a smaller number of edge pixels for EDR calculation. For the case of a plain or uniform background, the regions for EDR calculation also have quite a small number of edge pixels, and the EDR is at a lower level.

An adaptive threshold is proposed to detect tamper attacks without parameter adjustment for time-varying scene characteristics due to illumination or environmental changes, especially for nighttime video sequences with a lower number of edge pixels. The adaptive threshold for tamper detection in the *n^th^* frame is given as follows.

(3) $$Th_{n}=\{\begin{array}{ll} 150/E_{n}, & \text{if}\;\frac{E_{n}}{W\cdot H}<0.026\\ 400/E_{n}, & \text{if}\;0.026\leq \frac{E_{n}}{W\cdot H}<0.046\\ 1500/E_{n}, & \text{otherwise} \end{array}$$

where *E_n_*, *W* and *H* represent the number of edge pixels in the *n*-th background frame, the width and the height of a frame. The constants for the adaptive threshold are set empirically after the analysis of edge characteristics for various types of scene contents.

### 3.6. Tamper Decision

As illustrated in Figure 3, a tamper decision is made in the frame basis by using the EDR, the AEDR and the adaptive threshold of each frame. The decision process consists of tamper validation, tamper flag setting and tamper flag fixing.

To prevent wrong false alarms due to noise or temporary scene characteristics changes, a tamper validation count is defined as follows.

(4) $$S=\{\begin{array}{ll} \min(S+1,W), & \text{if}\;{\text{EDR}}_{n}\geq {\text{AEDR}}_{n}+Th_{n}\\ \max(S-1,0), & \text{otherwise} \end{array}$$

where *W* represents the size of the tamper validation window in frame interval units.

Finally, a tamper flag for the *n*-th frame is set after validation as follows.

(5) $$f_{n}^{t}=\{\begin{array}{cc} 1, & \text{if}\;S\geq T_{\text{set}}\\ 0, & \text{otherwise} \end{array}$$

where *T_set_* is a pre-configured interval for tamper validation. There exists a trade-off between *T_set_* and the false alarm rate. This value was empirically set at three frame intervals in our experiments.

If tamper attacks last longer than the learning period of the background generation by GMM, the tamper attacks get absorbed into the background. To prevent the release of the tamper flag due to long lasting tamper attacks, the tamper flag is fixed to a set state for tamper attacks lasting longer than the pre-configured period *T_fix_*. Once the tamper flag is fixed, only operators can clear it.

## 4. Experimental Results

### 4.1. Experimental Environments

To evaluate the performance of the proposed tamper detection algorithm, various surveillance video sequences in QVGA (320 × 240) resolution at 30 Hz were used in our experiments. The pre-configured periods *T_init_* and *T_fix_* were all set at 600 frame intervals. Since no commonly-used test sequences for camera tamper detection are available, we captured the test video sequences in the vicinity of our work place. A detailed description of the captured test sequences is given in Table 2.

**Figure 6:**
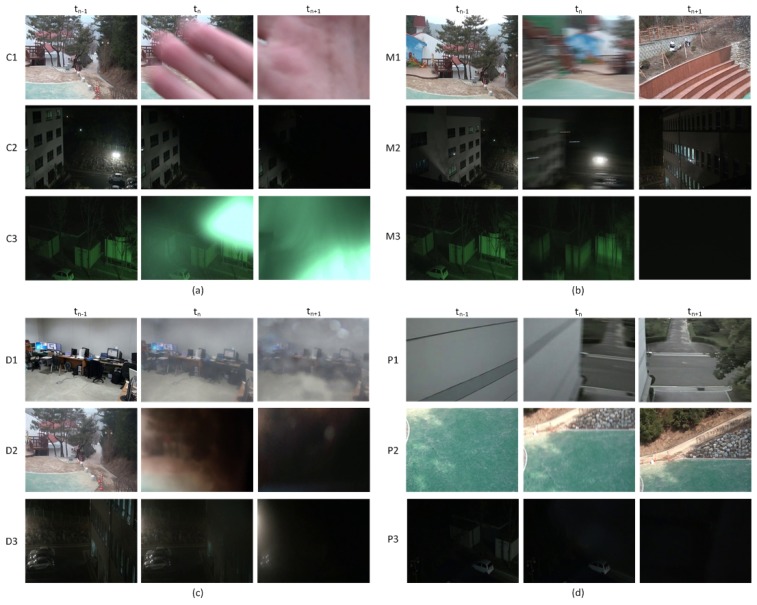
An example image for EDR calculation: (**a**) covered sequences; (**b**) moved sequences; (**c**) defocused sequences; (**d**) plain sequence (simple and plane background or very low illumination).

The test sequences include 48 daytime and 45 nighttime video sequences with camera tamper attacks and six video sequences without any attack. There are 10 covered, 23 moved and 15 defocused video sequences in the daytime sequences and 17 covered, 14 moved and 14 defocused video sequences in the nighttime sequences. Each sequence with camera tamper attacks includes at least one covered, moved or defocused event. Each test sequence is edited such that tamper attacks occur after Frame 600, which is the minimum time for stable background generation. Figure 6a–c shows some of the covered, moved and defocused video sequences used for performance evaluation.

In general, the background monitored by a surveillance camera is not so plain as the walls of a building or floors with a only few edges, as is shown in Figure 6d. In this study, the sequences with a simple background or with very low illumination light are called plain sequences. For plain sequences, the EDR is quite small compared to that of normal sequences due to the reduced number of edges, and thus, the detection performance will be degraded. Although plain sequences rarely exist in real video surveillance environments, some plain test sequences were used to evaluate the performance.

**Figure 7:**
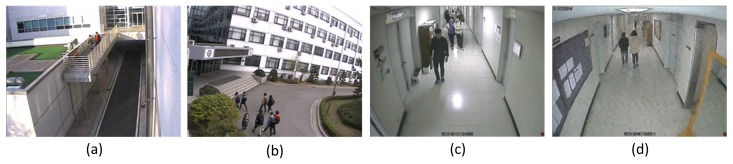
Surveillance sequences used for daily false alarm rate (DFAR) evaluation: (**a**) rooftop of a building with a wall; (**b**) main entrance of a building; (**c**) hallway with light reflection; (**d**) hallway with a mirror and noises.

The video sequences for false alarm performance evaluation, shown in Figure 7, include two 24-h outdoor sequences and four indoor daytime sequences with scene dynamics, such as moving objects, light reflection on the floor, quality degradation due to low video signal integrity, *etc*.

### 4.2. Performance Measures of Camera Tamper Detection

The accuracy of the proposed tamper detection algorithm is measured by the following detection rate, precision rate and daily false alarm rate with the terms defined in Table 3. The detection rate (DR) and the precision rate (PR) are defined as:

(6) $$DR=\frac{TP}{TP+FN}$$

(7) $$PR=\frac{TP}{TP+FP}$$

Additionally, the daily false alarm rate (DFAR) is defined as follows.

(8) $$\text{DFAR}=\frac{FP}{T}$$

where *T* represents the overall length of video sequences in the unit of days.

### 4.3. EDR and the Adaptive Thresholds for Different Scene Contents

**Figure 8:**
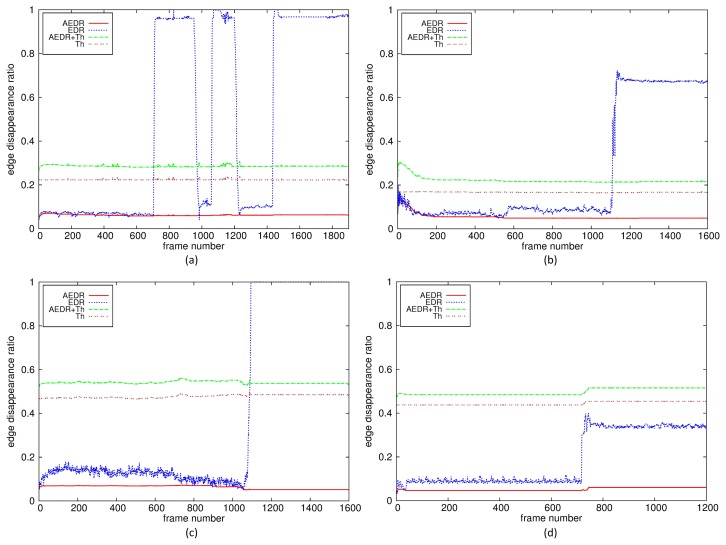
Tamper detection with the adaptive threshold for different scene contents: (**a**) daytime sequence, successive covered events; (**b**) daytime sequence, plain background; (**c**) nighttime sequence, defocused; (**d**) nighttime sequence, covered.

Figure 8 shows the EDR, the AEDR and the adaptive thresholds for three video sequences: a daytime sequence with successive covered events, a daytime plain sequence with a moved event and a nighttime sequence with a defocused event. These sequences have quite different scene characteristics, especially the number of edges in the background. The numbers of edges in the plain and the nighttime sequences are quite a bit smaller than that in the normal daytime sequence. However, the unified measure EDRs are shown to provide enough deviation from the AEDRs for all kinds of tamper detection in various environments. Furthermore, the adaptive thresholds for these sequences are shown to be at sufficient levels to robustly delineate the EDRs from the AEDRs for tamper attacks.

In Figure 8a, there are two short and one long covered tamper attacks. The first, the second and the third tamper attacks last 267, 158 and 1,460 frame intervals, respectively. The third attack represents the situation in which the tamper flag is fixed to a set state for an attack lasting longer than the pre-configured period. Once the tamper flag is set, the EDR continues to keep the recent value, and the difference between the EDR and the AEDR is still larger than the adaptive threshold, which prevents the tamper alert from being released due to background absorption of the tamper attacks.

Figure 8b,c shows the results when the proposed algorithm controls the adaptive threshold for a plain background or for nighttime video sequences with low illumination. Although the numbers of edges that disappear from the background after a tamper attack are quite a bit smaller than is the case in normal sequences with enough edges, shown in Figure 8a, the proposed algorithm can also detect this situation as a tamper attack by translating the scene characteristics into the edge disappearance rate in the background and by using the adaptive thresholds. Figure 8d shows the results when the proposed algorithm fails for the environments where the camera is not equipped with IR mode and there is little light. The EDR is not larger than the threshold because few edges are extracted due to very low illumination light. However, this is not common for a real video surveillance environment, where cameras have IR capability or illuminating light for nighttime. The proposed algorithm can detect tamper attacks for nighttime video sequences captured with IR mode in the same environment.

### 4.4. Tamper Detection Performance

The performance of the conventional and proposed camera tamper detection algorithms are summarized in Tables 4 and 5, respectively. All of the data in Table 4 are from the papers in which the algorithms were introduced.

For covered events in all daytime and nighttime sequences, the proposed algorithm shows a 95.8% detection rate and the same precision rate. For moved events, it shows a 97.3% detection rate and the same precision rate. The proposed algorithm detected defocused camera events with a detection rate of 96.7% and a precision rate of 93.5%. For all kinds of tamper attacks, the proposed algorithm shows a 96.5% detection rate and a precision rate of 95.7%. Compared with the detection accuracies of the conventional algorithms, shown in Table 4, the proposed algorithm has higher DR and PR for all types of tamper attacks, which proves that the proposed EDR and the adaptive threshold scheme can effectively reflect scene characteristics before and after tamper attacks very well. Although there is no difference in DR for daytime and nighttime sequences, PR in nighttime sequences is somewhat lower than that of daytime sequences. From the experimental results, the proposed algorithm is shown to be superior to most conventional algorithms in both DR and PR.

### 4.5. False Alarm Performance for 24-h Sequences

Most conventional studies have evaluated false alarm performance with very short video sequences with or without tamper attacks [8–10,12,14]; only two studies have evaluated false alarm performance with rather long sequences [11,13]. Short video sequences cannot reflect environmental changes, which may cause false alarms, or time-varying illumination conditions and scene dynamics altered by external disturbances, such as moving objects and the swaying of cameras or trees by wind.

Therefore, the authors propose using DFAR in Equation (8) for the evaluation of the false alarm performance. Table 6 shows the DFAR performance of the proposed algorithm for two 24-h sequences without tamper attacks and for the four less than one-hour sequences without tamper attacks shown in Table 2. The DFAR of the proposed algorithm is 0.47, quite a bit lower than those of the conventional studies, which means that there is less than one false alarm for two days. Excluding the DFAR in [8] due to the very short duration of its false alarm test sequences, the DFARs of the conventional studies handling all types of tamper attacks are larger than 106.67 [9,10], with a false alarm every 13.5 min, which is not acceptable for video surveillance systems.

## 5. Conclusions

A unified camera tamper detection algorithm that can detect all types of tamper attacks based on edge and object information is proposed. The edge disappearance rate of the background frame is defined as a unified measure of the deviation of the current frame from the background frame, excluding scene dynamics due to foreground objects. Tamper attacks are detected by comparing the difference between the EDR and the AEDR, with the adaptive threshold reflecting environmental conditions. The proposed algorithm outperforms most conventional studies in the detection rate and the precision rate for all types of tamper attack. The daily false alarm rate is sufficiently low for the proposed algorithm to be applied to real video surveillance systems, in which the DFARs in conventional studies are too high to be acceptable.

## Figures and Tables

**Table 1:** Parameters of Gaussian mixture model (GMM) in the proposed tamper detection algorithm.

**Parameters**	**Meaning**	**Value**
*K*	the number of mixture components	4
*σ*^2^	initial standard deviations of components	10
γ	learning rate	0.001
*ϖ*	mixture weight	0.05

**Table 2:** Video sequences for performance evaluation.

**Type**	**Number of Seq.**	**Illumination Condition**	**Overall Duration**
no-tamper (24 h)	2	day/night outdoor	48 h
no-tamper (1 h)	4	indoor	3.1 h
covered	27	indoor, day/night outdoor	17 m
moved	37	indoor day/night outdoor	22 m
defocused	29	indoor day/night outdoor	23 m

**Table 3:** Classification of the detection results.

**Term**	**Definition**
TP	alert for a tamper attack
TN	no alert for no tamper attack
FN	no alert for a tamper attack
FP	alert for no tamper attack

**Table 4:** Accuracy of the conventional tamper detection algorithms. PR, precision rate.

**Algorithm**	**Event**	**TP**	**FN**	**FP**	**N**	**DR**	**PR**	**DFAR**	Duration *
Saglam and Temizel [8]	covered	38	2	0	40	95.0%	100.0%		
	moved	11	1	0	12	91.7%	100.0%	0.00	14 m
	defocused	29	6	0	35	82.9%	100.0%		
Wang *et al.* [9]	*all*	56	1	2	57	98.2%	96.6%	106.67	27 m **
Kryjak *et al.* [10]	covered	16	0	4	16	100.0%	80.0%		
	moved	22	0	3	22	100.0%	88.0%	504.00	20 m **
	defocused	24	0	0	24	100.0%	100.0%		
Aksay *et al.* [11]	covered	20	0	13	20	100.0%	90.4%	196.00	6 h
	defocused	8	1	36	9	88.9%	77.2%		
Huang *et al.* [12]	covered	29	1	0	30	96.7%	100.0%		
	moved	28	2	4	30	93.3%	87.5%	N/A	N/A
	defocused	28	2	1	20	93.3%	96.6%		
Ribnick *et al.* [13]	covered	19	1	5	20	95.0%	79.2%	6.32	19 h
Alippi *et al.* [14], network	defocused	N/A	N/A	N/A	N/A	96.0%	84.0%	N/A	28 m
Alippi *et al.* [14], node	defocused	N/A	N/A	N/A	N/A	96.0%	76.0%	N/A	28 m

* Overall duration of sequences without tamper attack used for false alarm test;

** estimated from the literature.

**Table 5:** Tamper detection accuracy of the proposed algorithm.

**Time**	**Event**	**TP**	**FN**	**FP**	**N**	**DR**	**PR**
day	covered	18	1	0	19	94.7%	100.0%
	moved	22	1	0	23	95.7%	100.0%
	defocused	16	0	1	16	100.0%	94.1%

	sub-total	56	2	1	58	96.6%	98.2%

night	covered	28	1	2	29	96.6%	93.3%
	moved	14	0	1	14	100.0%	93.3%
	defocused	13	1	1	14	92.9%	92.9%

	sub-total	55	2	4	57	96.5%	93.2%

Total		111	4	5	115	96.5%	95.7%

**Table 6:** Daily false alarm rate of the proposed algorithm.

**Type**	**Overall Duration**	**Number of False Alarms**	**DFAR**
no-tamper (24 h)	48 h	0	
no-tamper (1 h)	3.1 h	1	

Total	51.1 h	1	0.47
